# A young man with exertional chest discomfort

**DOI:** 10.1007/s12471-019-1275-9

**Published:** 2019-04-11

**Authors:** L. E. Lezcano Gort, B. Roque Rodríguez, M. R. Porro Fernández

**Affiliations:** Department of Cardiology, San Pedro de Alcantara Universitary Hospital, Cáceres, Spain

In this case we present a 43-year-old athletic male, nonprofessional cyclist with unremarkable medical history. He had been admitted to the emergency department because of chest discomfort, dizziness, heavy sweating and nausea after cycling 40 km in extreme heat (local temperature 41.5 degrees Celsius). Physical examination revealed a Glasgow Coma Scale score of 15, and he was haemodynamically stable. The laboratory results showed: creatine kinase 870 IU/l, creatine kinase-MB 12 IU/l, troponin T 16 ng/ml, and creatinine 1.6 mg/dl. Out-of-hospital electrocardiogram taken at first medical contact is shown in Fig. 1.

1. What is your diagnosis?
2. What is the most likely mechanism and the suggested therapy?

**Fig. 1:**
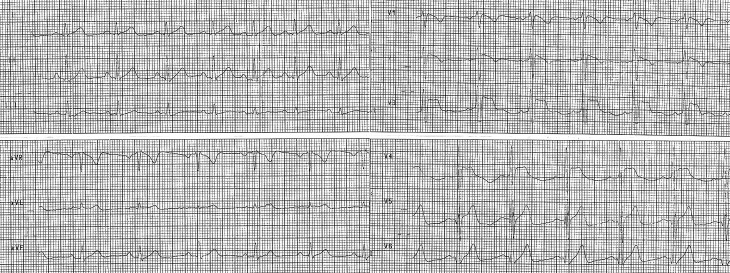
Out-of-hospital electrocardiogram at first medical contact

## Answer

You will find the answer elsewhere in this issue.

